# Cellular Midpalatal Suture Changes after Rapid Maxillary Expansion in Growing Subjects: A Case Report

**DOI:** 10.3390/ijms18030615

**Published:** 2017-03-11

**Authors:** Alberto Caprioglio, Rosamaria Fastuca, Piero Antonio Zecca, Matteo Beretta, Carlo Mangano, Adriano Piattelli, Aldo Macchi, Giovanna Iezzi

**Affiliations:** 1 Department of Surgical and Morphological Sciences, University of Insubria, 21100 Varese, Italy; alberto.caprioglio@uninsubria.it (A.C.); pieroantonio@gmail.com (P.A.Z.); teoberet@libero.it (M.B.); aldo.macchi@uninsubria.it (A.M.); 2 Department of Medical, Surgical and Health Sciences, University of Messina, 98100 Messina, Italy; 3 Department of Medical, Oral and Biotechnological Sciences, University of Chieti-Pescara, 66100 Chieti, Italy; camangan@gmail.com (C.M.); apiattelli@unich.it (A.P.)

**Keywords:** suture, maxillary expansion, treatment

## Abstract

The present case report aimed to investigate immediate histologic changes in midpalatal suture in humans following rapid maxillary expansion compared to control. Three patients (mean age 8.3 ± 0.9 years) were enrolled in the case report and underwent midpalatal suture biopsy. Two patients underwent treatment before biopsy. The third patient did not show transversal maxillary deficiency and was enrolled as a control. Biopsy samples of midpalatal suture at 7 (subject 1) and 30 days (subject 2) after maxillary expansion as well as of one control (subject 3) were collected and processed for histology. In the control (subject 3) inter-digitations at the palatal suture gap were observed. At 7 days (subject 1) mature bone with small marrow spaces and trabecular bone with the peculiar storiform appearance inside the soft tissue and collagen fibers running parallel only in the central part were present. At 30 days (subject 2), a greater number of newly-formed bone trabeculae with a perpendicular orientation to the long axis of the suture could be seen. At 30 days the fibrous component of bone tissue was less represented compared to the sample at 7 days. Data from the preliminary histological results showed that bone formation was observed in the gap after rapid maxillary expansion, although the healing process was still ongoing.

## 1. Introduction

Maxillary expansion is a valid procedure to correct transversal upper arch deficiency [1,2] exerting a force along the midpalatal suture, which was previously described as the growth site [3]. Rapid maxillary expansion (RME) protocols are most commonly performed by clinicians. Indeed, RME might be indicated for a variety of clinical conditions that orthodontists routinely treat, as recently reported [4]. Reduced transverse growth of the maxilla might lead to sagittal problems and underdevelopment of the midface [5]. Maxillary constriction might be also associated to occlusal disharmony and esthetics as well as such functional problems involving breathing pattern anomalies [6,7,8,9]. RME was indicated as an effective procedure in order to increase maxillary transverse discrepancy but with various positive side effects on general health of growing patients, increasing its number of indications [4]. The main orthodontic outcome of RME is an increase in the upper arch transverse dimensions, due to skeletal and dental changes. The effect of treatment has been shown to be midpalatal suture separation with the defect then created reported to be rapidly filled with new bone [10]. Dimensional changes in the midpalatal suture produced by RME in growing subjects have been investigated by conventional radiographic techniques [11] and cone beam computed tomography (CBCT) [12,13]. Radiographic studies showed significant density reduction along the midpalatal suture at the end of active expansion and an increase in sutural density after 6 months of retention, indicating reorganization of the midpalatal suture [14]. Along with radiographic investigations, morphologic and histologic studies in animals have been performed in order to assess changes in the midpalatal suture due to expansion procedures. Histologic investigations in animals showed that mechanical separation at the midpalatal suture gained by RME is a multiple step healing process, characterized by new bone and connective tissue formation, followed finally by remodeling [15,16,17,18]. Remodeling seems to be continuous, but Storey [15] reported that even after 3–4 weeks the normal serrated inter-digitated form of suture had not yet been reconstituted [15]. Melsen [19,20] performed the first and only investigation on human beings by collecting bioptic samples at the midpalatal sutures of eight children ranging from 8 to 13 years of age, at various stages during and following RME, starting from 3 weeks after the end of the active expansion phase with autoptic samples as controls. Melsen [17] found that a true stimulation of sutural growth was reported only in children before the pubertal growth spurt. When the patient become older several microfractures in the sutural region were found, influencing healing processes and preventing further growth within the midpalatal suture. Deepening knowledge of histologic response to RME treatment and healing processes is indeed helpful in order to better understand the timing for starting treatment since younger patients seemed to have a more favorable response. Moreover, important information might be derived from the investigation of healing time and phases in order to be oriented in choosing correct retention time intervals and preventing relapse. To our best knowledge, only Melsen [20] investigated these important aspects in human beings, arising then the need for further confirmation of previously reported results. Furthermore, considering that a long time has passed since the publication of the previously cited article, an update might be useful. In any case, Melsen [20] did not enroll bioptic controls since the investigation used autopsy material as the control. The present case report therefore aimed to investigate changes in midpalatal suture in humans 7 and 30 days following RME with bioptic control samples.

## 2. Results

### 2.1. Histological Results

#### 2.1.1. Control (Subject 3, Male)

Mature bone with small marrow spaces was observed. In the middle of the biopsy a palatal suture gap was detected, characterized by inter-digitations.

The width of the palatal suture ranged from 194.2 μm to 470.1 μm, depending on the analyzed segment. Remodeling of the bone margins of the palatal suture was seen, and the bone exhibited different maturation stages. In some fields, mature bone with small osteocyte lacunae was present, whereas in other areas, newly-formed bone, with wide osteocyte lacunae and a high affinity for the dyes, in particular acid fuchsin, could be seen.

The suture margins were irregular, and, in the inner part, connective tissue with several stromal cells could be found.

**Figure 1:**
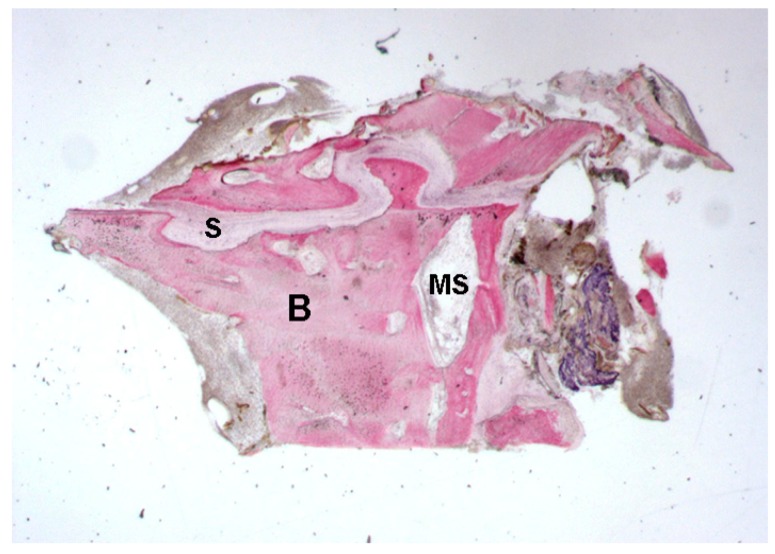
Control. Mature bone (**B**) with small marrow spaces (**MS**) was observed. The gap of the palatal suture (**S**) appeared characterized by inter-digitations. Toluidine blue and acid fuchsin were used. Original magnification 12×.

**Figure 2:**
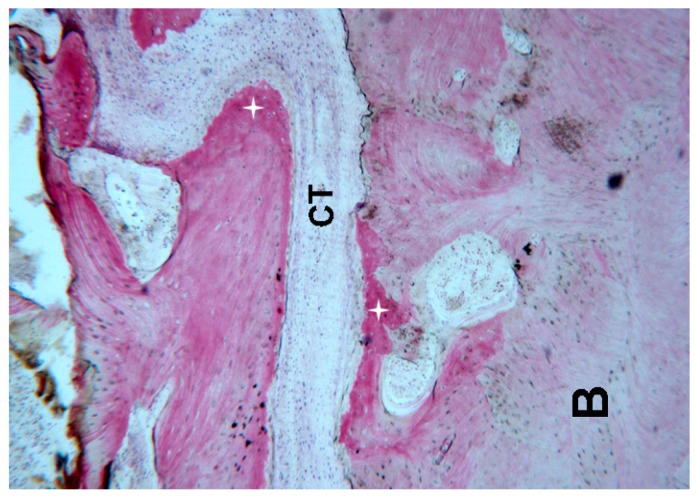
Control. Connective tissue (**CT**) in the middle of the suture as well as mature bone (**B**) and newly formed bone (**+**) was observed. Toluidine blue and acid fuchsin were used. Original magnification 40×.

**Figure 3:**
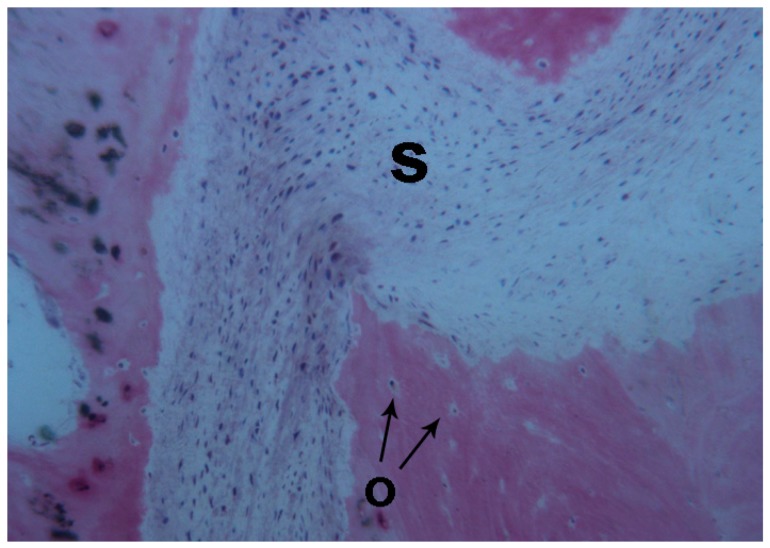
Control. The bone margins of the suture (**S**) were irregular. New bone with wide osteocyte lacunae (**O black arrows**) was observed. Toluidine blue and acid fuchsin were used. Original magnification 100×.

**Figure 4:**
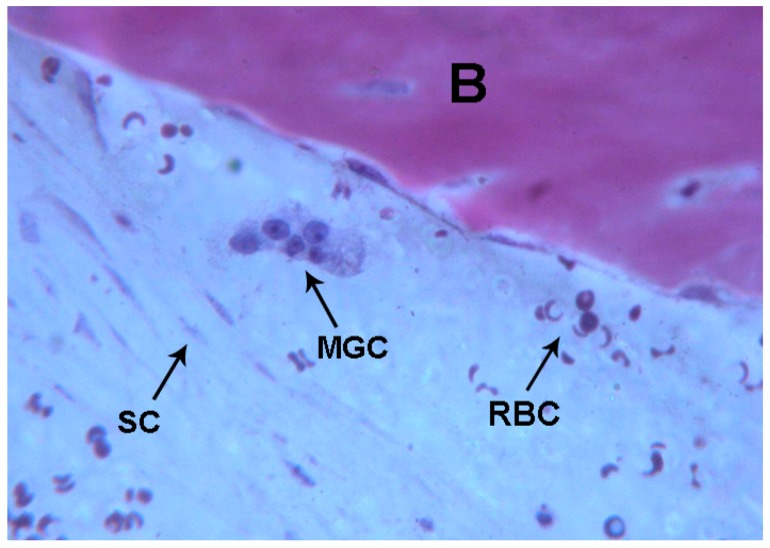
Control. Multi-nucleated giant cells (**MGC**) near the marginal bone, spindle cells (**SC**) and red blood cells were observed (**RBC**). Bone (**B**).Toluidine blue and acid fuchsin were used. Original magnification 400×.

At higher magnification, a multi-nucleated giant cell in the vicinity of the bone margin was seen. Moreover, in the same area, spindle cells and red blood cells were evident, probably also due to the surgical trauma (Figure 1, Figure 2, Figure 3 and Figure 4).

#### 2.1.2. Test 7-Days (Subject 1, Female)

**Figure 5:**
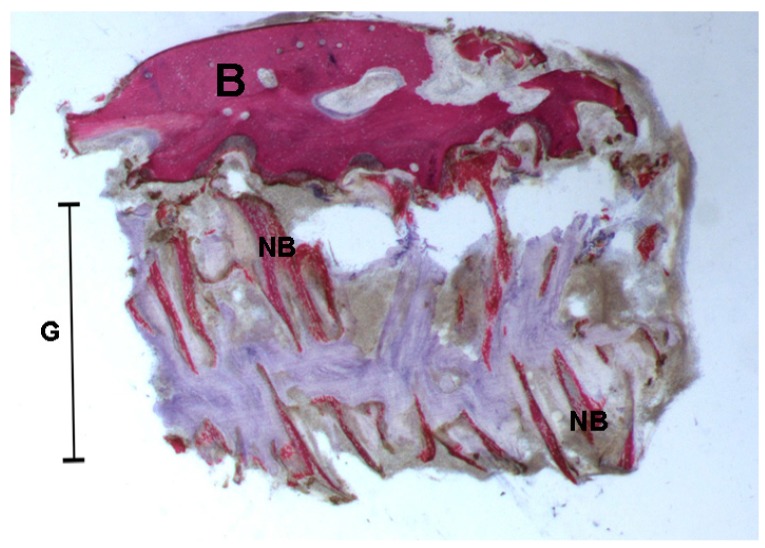
Mature bone with small marrow spaces (**B**) and, in the gap (**G**) after rapid maxillary expansion, trabecular new bone (**NB**) with storiform appearance was observed. Toluidine blue and acid fuchsin were used. Original magnification 12×.

Mature bone with small marrow spaces and trabecular bone with the peculiar storiform o fishbone appearance inside the soft tissue was observed (Figure 5).

**Figure 6:**
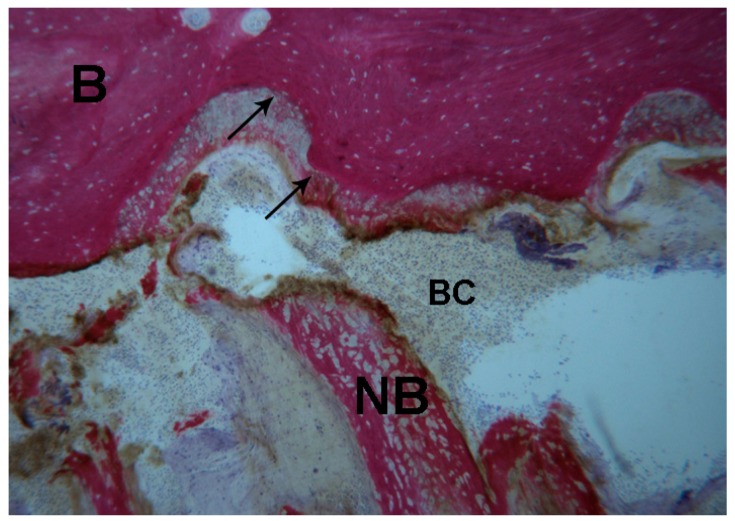
Test 7 days. The bone margins of the suture were characterized by inter-digitations (**black arrows**), where the newly-formed osteoid matrix undergoing mineralization directly was also observed. Inside the suture, newly-formed trabecular bone (**NB**) was detected. In the suture gap a blood clot (**BC**) was present. Toluidine blue and acid fuchsin were used. Original magnification 40×.

**Figure 7:**
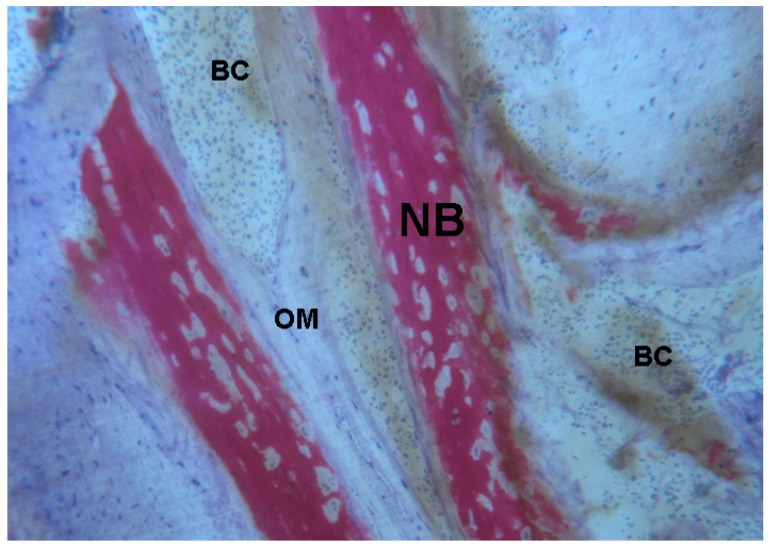
Test 7 days. The trabecular newly formed bone (**NB**) had a parallel trend and was surrounded by osteoid matrix (**OM**) undergoing mineralization. In a few areas of the blood clot (**BC**) numerous red blood cells were present. Toluidine blue and acid fuchsin were used. Original magnification 100×.

**Figure 8:**
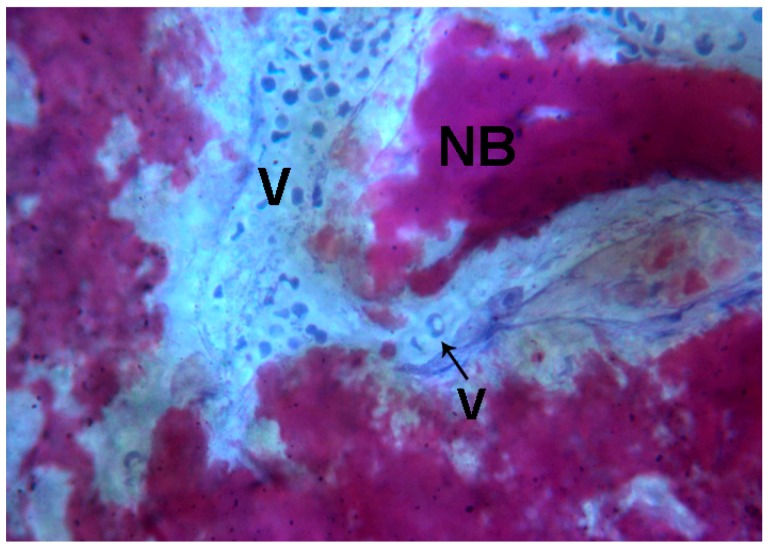
Test 7 days. Newly-formed spicules (**NB**) close to large (**V**) and small vessels (**V and black arrow** were observed. Toluidine blue and acid fuchsin were used. Original magnification 200×.

After the rapid maxillary expansion, the bone margins of the suture were characterized by inter-digitations. In many fields, it was possible to observe newly-formed bone and newly-formed osteoid matrix undergoing mineralization directly on the margins of the suture. Inside the suture, after rapid maxillary expansion, observed newly-formed bone with trabeculae running parallel was observed, that were not in contact with the pre-existing bone and that were surrounded by osteoid matrix undergoing mineralization. Within the suture, a few areas of blood clot with several red blood cells were observed, probably caused by the trauma of the mechanical separation at the midpalatal suture. The connective tissue was populated by stromal cells. In some fields, newly-formed spicules of bone undergoing mineralization close to small and large blood vessels were observed (Figure 6, Figure 7 and Figure 8). Histomorphometry showed 14% newly-formed bone and 86% soft tissue.

#### 2.1.3. Test at 30 Days (Subject 2, Male)

As with the sample at day 7, several newly-formed bone trabeculae aligned parallel to each other with a perpendicular orientation to the long axis of the suture could be observed. In contrast to the sample from day 7, the parallel-oriented trabeculae were in a greater number and closer to each other, and in few fields, they merged into one another.

At high power magnification, intense osteoblastic activity and many areas with osteoid matrix undergoing mineralization were detectable, indicating a still-ongoing neoformation process. Only a small portion of the bone margins of the palatal suture was evident in the histological sample and it was lined by osteoblasts actively producing osteoid matrix.

**Figure 9:**
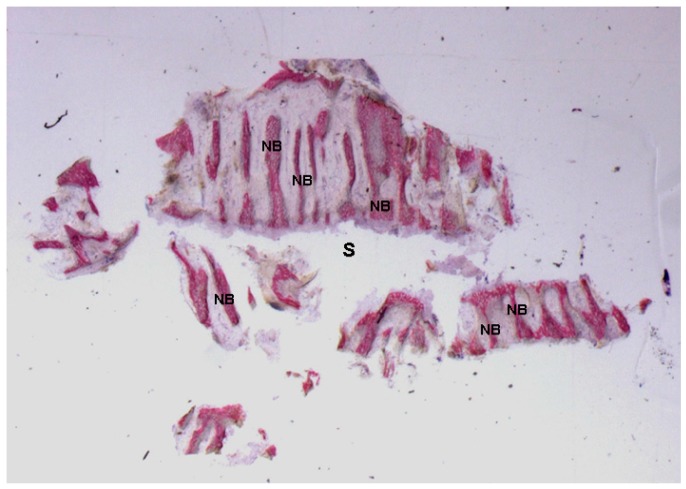
Test at 30 days. The newly-formed bone trabeculae (**NB**) were oriented perpendicularly to the long axis of the suture (**S**) and run parallel to each other. Toluidine blue and acid fuchsin were used. Original magnification 9×.

**Figure 10:**
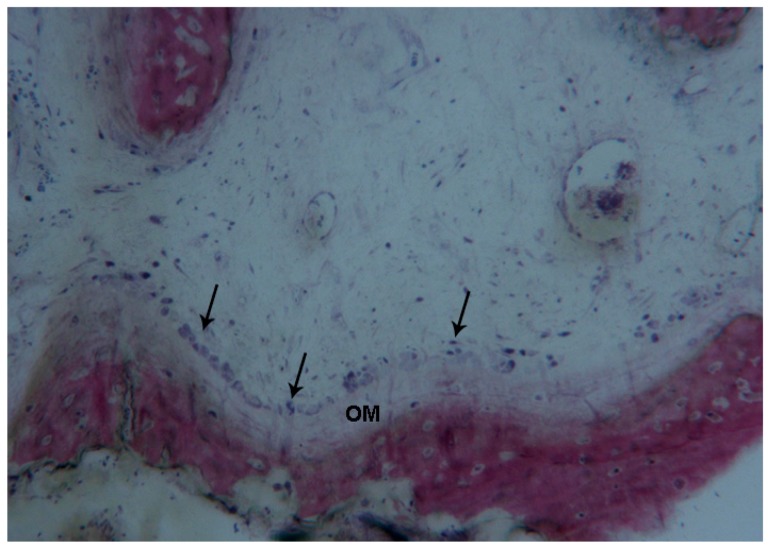
Test at 30 days. A small portion of the bone margins of the palatal suture was evident, lined by osteoblasts (**black arrows**) producing osteoid matrix (**OM**). Toluidine blue and acid fuchsin were used. Original magnification 100×.

**Figure 11:**
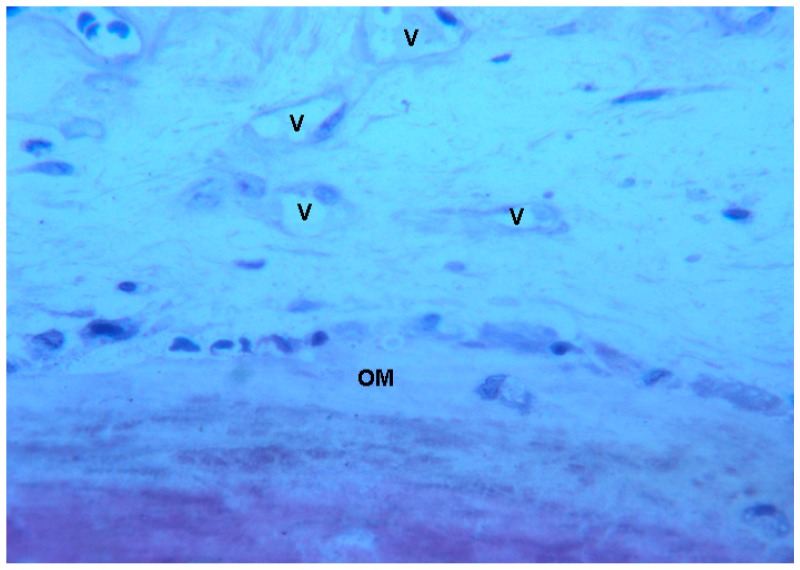
Test at 30 days. In the marrow spaces, several small blood vessels (**V**) in the vicinity of not yet mineralized osteoid matrix (**OM**) were present. Toluidine blue and acid fuchsin were used. Original magnification 200×.

In the vicinity of trabecular bone, in the marrow spaces, several small and large blood vessels, stromal cells and some lymphocytes were present (Figure 9, Figure 10 and Figure 11).

Histomorphometry showed 43% newly-formed bone and 57% soft tissue.

### 2.2. Polarized Light

#### 2.2.1. Control (Subject 3, Male)

**Figure 12:**
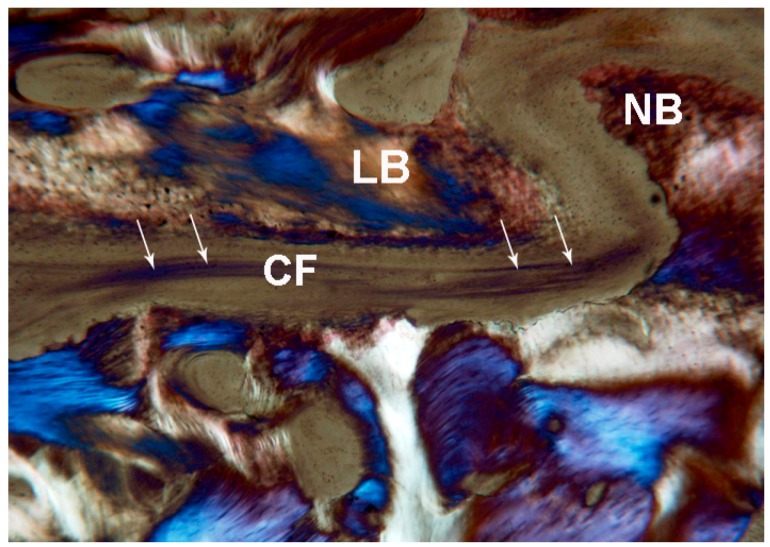
Control. Under polarized light, parallel lamellar bone (**LB**) and newly-formed bone (**NB**) without parallel collagen fiber orientation were observed. In the middle of the suture collagen fibers (**CF white arrows**) oriented parallel to the long axis of the suture were seen. Toluidine blue and acid fuchsin were used. Original magnification 40×.

Lamellar bone with collagen fibers with a parallel orientation was detected. In some fields of the marginal portion of the bone of the palatal suture, newly-formed bone without parallel-oriented collagen fibers was observed. The connective tissue found in the middle of the suture presented collagen fibers with a parallel orientation to the long axis of the suture (Figure 12).

#### 2.2.2. Test at 7 Days (Subject 1, Female)

**Figure 13:**
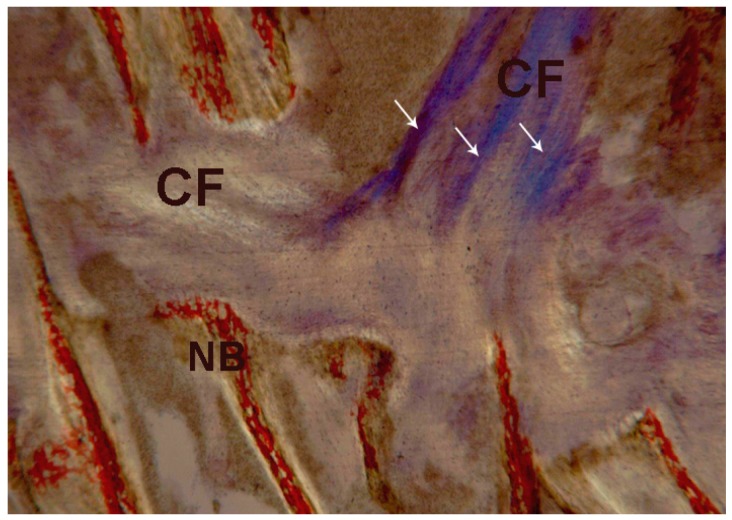
Test at 7 days. Under polarized light, newly-formed bone (**NB**) and collagen fibers (**CF**) with a storiform orientation (**white arrows**) were observed. Toluidine blue and acid fuchsin were used. Original magnification 40×.

Newly-formed bone without collagen fibers with a parallel orientation was observed. The connective tissue showed a storiform pattern and the collagen fibers were parallel only in the central part (Figure 13).

#### 2.2.3. Test at 30 Days (Subject 2, Male)

**Figure 14:**
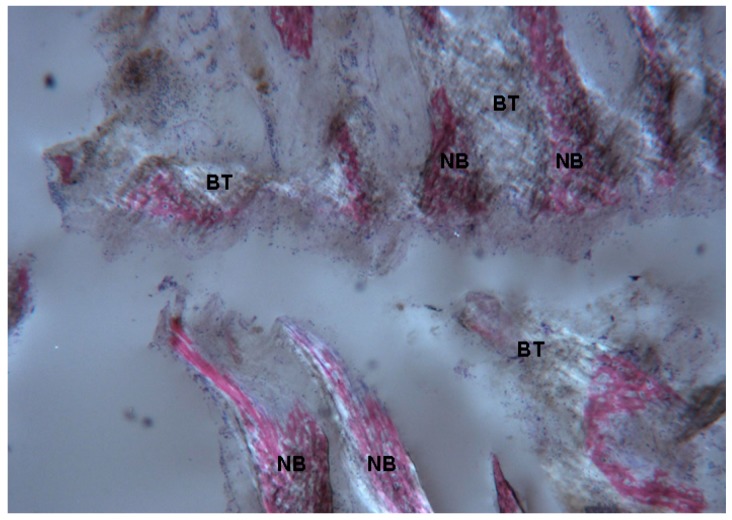
Test at 30 days. Under polarized light, newly-formed bone (**NB**) undergoing mineralization could be observed. Not yet organized newly-formed bone trabeculae (**BT**) were also evident. Toluidine blue and acid fuchsin were used. Original magnification 40×.

The bone tissue was not mature, with collagen still poorly organized. Only in a limited number of bone trabeculae was the bone tissue better organized, as collagen fibers started to be aligned in a parallel fashion. Generally, at 30 days the fibrous component of bone tissue was less represented compared to the sample at 7 days (Figure 14).

## 3. Discussion

Midpalatal suture changes after RME were reported to be of great interest [21] since a deeper knowledge of this processes might help in treatment options and modalities. The present case report aimed to investigate immediate histologic changes in midpalatal suture in humans following RME compared to control and was conducted on selected patients who had to perform surgery in the palatal area for other clinical reasons. A similar previous investigation did not enroll biopsy controls [20] which were instead enrolled in the present case report. Despite the young age (8.3 ± 0.9 years), the subjects exhibited suture gap but with inter-digitations in the midpalatal suture, as previously reported in patients around the age of 10 [20]. At this age, changes in suture morphology from squamosity to sinuosity were observed [20], supporting the present report since remodeling of the bone margins of the palatal suture was evident with different maturation stages of the newly-formed bone areas characterized by wide osteocyte lacunae. Moreover, the morphology of the suture at this age (subject 3) presented a parallel orientation of the collagen fibers related to the suture long axis similar to lamellar bone, which is traditionally considered as a stress-strain resistant type of bone. RME performed in pre-pubertal age might avoid fracture of inter-digitations due to immature stages of growth and bone remodeling [21]. Seven days after the end of expansion (subject 1) newly-formed bone with osteoid matrix undergoing mineralization was evident not only on the bone margins, indicating mineralization processes from within the center of the suture, which seemed to be an original finding of the present investigation, as was the peculiar fishbone appearance of the trabecular bone. Moreover, newly-formed bone showed collagen fibers in a transversal orientation related to the suture long axis in comparison to the control sample, where a longitudinal orientation was observed. This orientation was suggested to be related to the response to mechanical forces as shown in mice and this finding has never been reported in humans [22]. Within the suture few areas of blood clot with several red blood cells were observed, probably caused by the trauma of maxillary expansion, but no inflammation cells were reported. This result is in contrast with Melsen [20] who reported hyperemia and inflammation associated with osteoblastic activity near to the bone margins in the midpalatal suture 3 weeks after expansion. Inflammation cells were not evident at 7 and 14 days after expansion also in several animal investigations, meanwhile the osteoblastic activity was reported right after the midpalatal splitting [17]. Cleall et al. [16] found predominantly disruptive, inflammatory processes and osteoclastic activity in monkeys. The initial inflammatory aspect was reported only in these investigations, meanwhile the hyperemia seemed to be present in all the performed studies on animals [15,17] and human beings [20] in the first few days after midpalatal suture separation. In the present case report no inflammation cells nor osteoclastic activity was noted. Although inter digitations were present in the suture at this stage of growth the suture morphology was still immature. At 30 days (subject 2) there was a 29% increase of newly-formed bone which showed thicker trabeculae and the peculiar fishbone appearance with parallel orientation and confluent in several areas. Diffused osteoblastic activity was evident also at this stage but also vascular activity was noted. Similar results were reported by Melsen [20] who found newly-formed bone along the margins and in the middle of the suture with confluent islands in cross-section bony extensions 4 weeks after the end of expansion but no angiogenesis was reported and the normal serrated inter-digitated shape of suture had not yet been reconstituted. The results of the present investigation are limited to the restricted number of patients, since bone biopsy is not ethically justified only to assess treatment outcomes. Therefore, only patients who had to undergo surgery for clinical reasons could be selected. Furthermore, the results are limited to the appliance that was tooth-anchored on primary teeth without any other skeletal anchorage [23]. Moreover, the observation time was limited to 30 days after RME but it would be very useful to perform long-term investigations. The quantity of RME might affect the suture since rapid expansion protocols were shown to create midpalatal suture separation followed by the filling of the defect with new bone. Once the midpalatal suture separation is obtained, the amount of expansion might affect the healing time of the bone that starts after the midpalatal separation and keeps going for several months after. The sample harvested from subject 2 showed more advanced healing processes compared to subject 1. Further case reports should be focused on the investigation in different timepoints in order to better clarify when the bone healing process might end and give important guidelines on retention and relapse.

## 4. Materials and Methods

### 4.1. Subjects

This case report enrolled subjects seeking orthodontic treatment and who had never been treated before, presenting at the Division of Orthodontics (University of Insubria, Varese, Italy). As a routine procedure, a signed informed consent for releasing diagnostic records for scientific purposes was obtained from the parents of the patients prior to entry into the treatment. The protocol was reviewed and approved by the local Ethical Committee (n${}^{\circ }$826 University of Insubria, Varese, Italy 10/08/2013) and the procedures followed adhered to the World Medical Organization Declaration of Helsinki. Inclusion criteria were: (1) good general health according to medical history and clinical judgment [24,25,26]; and (2) presence of mesiodens palatally located in the anterior portion of maxillary bone which needed to be surgically removed since they were causing alterations in the position of permanent upper incisors or their retention. Three patients (1 female and 2 males, mean age 8.3 ± 0.9 years) were enrolled in the case report. Two patients (1 female, subject 1 and 1 male, subject 2) presented unilateral posterior crossbite [27] and underwent RME treatment before mesiodens extraction in order to facilitate surgical procedure by reducing bone resistance during bone remodeling after suture expansion. The third patient did not show transversal maxillary deficiency but a mesiodens was present. This patient was enrolled as control (subject 3). All the three enrolled patients were skeletal Class I with normal overbite and overjet. None of them presented craniofacial asymmetries, pathological alterations or syndromes. Each patient underwent CBCT recordings (i-CAT, Imaging Sc. Int., Hatfield, PA, USA) performed in seated position (120 kV, 3.8 mA, 30 s) [28,29,30] prior to the surgical treatment to accurately plan the surgery. The expander used was the Hyrax type expander (10-mm screw, A167-1439, Forestadent, Pforzheim, Germany) banded to the upper second deciduous molars. The screw of the palatal expander was initially turned two times (0.45 mm initial transversal activation). Afterwards, parents of the patients were instructed to turn the screw once each following day (0.225 mm activation per day). The maxillary expansion was performed until dental overcorrection. The screw was then locked with light-cure flow composite (Premise Flowable; Kerr Corporation, Orange, CA, USA) and the expander was kept on the teeth as a passive retainer. During this period, none of the patients underwent any further orthodontic treatment. The amount of expansion was 5.6 mm for subject 1 and 4.7 mm for subject 2. The amount of expansion was confirmed by measuring the opening of the expansion screw with the mean of a manual intraoral caliper. No X-rays were acquired after expansion treatment and the effect of the appliance was confirmed with the opening of upper central incisors diastema clinically documented in both subject 1 and 2 and the correction of the crossbite until dental overcorrection (defined as when lingual cusps of upper first molars occluded onto lingual side of buccal cuspids of the lower first molars).

### 4.2. Biopsy Procedure of the Midpalatal Suture

The biopsy of the midpalatal suture was performed the same day of surgery for the removal of the mesiodens in each subject. The biopsy was performed before the removal of the pathologic tissue to avoid contamination. After the mucous membrane of the hard palate was raised, a cylindrical trephine bur 7 mm in diameter was used to perform the biopsy. The bone biopsy was located on the midline along the midpalatal suture and slightly moved towards the right or left to involve suture tissue and one-side bone margin. The sample was taken anterior or posterior to the nose–palatal foramen according to the position of the mesiodens, in order to remove bone in correspondence of the further surgical site. The bone at that level would be removed with subtraction by the mean of the burs in order to expose and extract the pathologic bulk. This removal was in these cases performed with a trephine bur in order to collect samples for the case report. Subjects 1 and 2 underwent RME treatment and subject 3 was used as control and underwent no treatment. Each subject underwent only one biopsy and they were performed at different timepoints in different patients. Subject 1 underwent biopsy 7 days after RME and subject 2 underwent biopsy 30 days after RME. The control patient (subject 3) underwent surgery for mesiodens extraction and then midpalatal suture biopsy after diagnosis since no other treatments were needed.

### 4.3. Histological Processing

The specimens retrieved from the palatal suture were processed to obtain thin histologic slides. They were dehydrated in a graded series of ethanol rinses and embedded in a glycolmethacrylate resin (Technovit 7200 VLC, Kulzer, Wertheim, Germany). After polymerization, the specimens were sectioned along their longitudinal axis, with a high-precision diamond disk at about 150 μm and ground down to about 30 μm with a specially designed grinding machine Precise 1 Automated System (Assing, Rome, Italy). Three slides were obtained from each specimen. These slides were stained with acid fuchsin and toluidine blue. Histomorphometry of the percentages of newly-formed bone and soft tissue was conducted using a light microscope (Laborlux S, Leitz, Wetzlar, Germany) connected to a high-resolution video camera (3CCD, JVC KY-F55B, JVC^®^, Yokohama, Japan) and interfaced to a monitor and PC (Intel Pentium III 1200 MMX, Intel^®^, Santa Clara, CA, USA). This optical system was associated with a digitizing pad (Matrix Vision GmbH, Oppenweiler, Germany) and a histomorphometry software package with image capturing capabilities (Image-Pro Plus 4.5, Media Cybernetics Inc., Rockville, MD, USA). Birefringence was measured as an indicator of transverse collagen orientation using polarized light. Collagen fibers were viewed by placing the thin sections of bone under an Axiolab light microscope (Laborlux S, Leitz, Wetzlar, Germany) equipped with two linear polarizers and two quarter wave plates arranged to have a transmitted circularly polarized light. Collagen fibers aligned perfectly transverse to the direction of the light propagation (parallel to the plane of the section) appeared bright due to a change in the refraction of existing light, whereas the collagen fibers aligned along the axis of light propagation (perpendicular to the plane of the section) appeared dark because no refraction occurred [31,32].

## 5. Conclusions

Within the limits of the present case report, and specifically the restricted number of patients due to the difficulty in ethically justifying bone biopsies to assess treatment outcomes and therefore the necessity to select patients who had to undergo surgery for clinical reasons, it can be concluded that 7 days after RME, initial bone neoformation was evident in the suture gap, whilst at 30 days it resulted half-filled with newly-formed bone tissue. Therefore, it would be very interesting to perform long-term investigations in order to have a better knowledge of palatal suture healing and therefore tune the timing of RME treatment.

## Abbreviations

RME	Rapid Maxillary Expansion
CBCT	Cone Beam Computer Tomography
